# Trachoma Impact Survey Results from 31 Woredas in Tigray Region, Ethiopia

**DOI:** 10.1080/09286586.2024.2317823

**Published:** 2024-12-27

**Authors:** Gemechis Teferi, Harnet Adane, Evini Cyrille, Aynalem Tefera, Solomon Gadisa, Adugna Amin, Mebratu Tsehaye, Yonas Mitku, Haftamu Assefa, Sharone Backers, Addisu Alemayehu, Belete Mengistu, Fikreab Kebede, Fentahun Tadesse, Nebiyu Negussu, Robert Butcher, Ana Bakhtiari, Rebecca Willis, Sarah Boyd, Cristina Jimenez, Michael Dejene, Anthony W. Solomon, Meheret Deyassa, Mohammed Shafi, Tezera Kifle, Asfaw Tegen, Berihu Mesfin, Tsegay Berihu, Teklay Mariam, Hagos Godefay, Emma M. Harding-Esch, Amanuel Kidane, Ephrem Fisseha

**Affiliations:** a Light for the World, Addis Ababa, Ethiopia; b Tigray Regional Health Bureau, Mekele, Tigray, Ethiopia; c Quha Hospital, Tigray, Ethiopia; d Act to End NTDs East, RTI International, Addis Ababa, Ethiopia; e Ministry of Health, Addis Ababa, Ethiopia; f Crown Agents, Addis Ababa, Ethiopia; g Clinical Research Department, London School of Hygiene & Tropical Medicine, London, UK; h International Trachoma Initiative, Task Force for Global Health, etc., Decatur, Georgia, USA; i Sightsavers, Haywards Heath, UK; j Sightsavers, Addis Ababa, Ethiopia; k Department of Control of Neglected Tropical Diseases, World Health Organization, Geneva, Switzerland; l Ambo Hospital, Ambo, Oromia, Ethiopia; m Adama Hospital Medical College, Adama, Oromia, Ethiopia; n Orbis International, Addis Ababa, Ethiopia; o Eyen Consulting PLC, Addis Ababa, Ethiopia

**Keywords:** Ethiopia, neglected tropical disease, Tigray, trachoma, trachoma impact survey

## Abstract

**Purpose:**

Baseline surveys were conducted in Tigray region, Ethiopia, in 2013. Since then, rounds of azithromycin mass drug administration (MDA) have been delivered in-line with international guidance. The purpose of these surveys was to assess trachomatous inflammation—follicular (TF) prevalence following those treatments to enable the region to plan the next steps towards elimination of trachoma.

**Methods:**

All surveys followed WHO recommendations for community-based cross-sectional survey design. Thirty-one woredas in six zones of Tigray region were surveyed. There were two survey series: all 31 woredas were surveyed in the first series, and 11 woredas were resurveyed in the second, due to having a TF prevalence between 5% and 9.9% in the first series.

**Results:**

In the first series of 31 surveys, one woreda had an adjusted TF prevalence in 1−9-year-olds of <5.0%, 13 had a prevalence of 5.0−9.9% and 17 had a prevalence of 10.0−29.9%. In the second series of 11 surveys, the prevalence of TF was <5.0% in seven woredas and 5.0−9.9% in four woredas. The most recent adjusted prevalence of trachomatous trichiasis (TT) unknown to the health system in ≥15-year-olds was ≥.2% in 27 EUs. One-third of households visited had access to an improved drinking water source within a 30-minute return journey of their house, and 11% had an improved latrine.

**Conclusion:**

Eight woredas met the criteria to stop MDA for 2 years before the re-survey. However, further rounds of MDA, additional efforts to improve water and sanitation access and ongoing strengthening of surgical services for TT are needed across Tigray.

## Introduction

Trachoma, caused by repeated rounds of infection with *Chlamydia trachomatis*, is the leading infectious cause of blindness worldwide.^1^ Trachoma can be eliminated as a public health problem using the World Health Organization’s (WHO)-recommended SAFE (trichiasis **s**urgery, mass drug administration [MDA] of **a**ntibiotics, **f**acial cleanliness, and **e**nvironmental improvement) strategy.^2,3^ Elimination of trachoma as a public health problem is defined as follows: (i) prevalence of trachomatous trichiasis (TT) unknown to the health system of <.2% among people aged ≥15 years in each formerly endemic district, (ii) prevalence of trachomatous inflammation—follicular (TF) of <5% among children aged 1–9 years in each formerly endemic district, plus (iii) evidence that the health system can continue to identify and manage incident cases of TT.^4^

In some parts of Ethiopia, large-scale trachoma elimination efforts have been underway since at least 2001.^5^ Pre-intervention (baseline) surveys, conducted both before and as part of the Global Trachoma Mapping Project,^6,7^ confirmed that trachoma was hyperendemic throughout much of the country,^8–14^ and stimulated stakeholders at all levels to take action.^15^ In the Tigray region, trachoma was endemic in all 11 evaluation units (EUs) in 2013,^16,17^ with disease prevalence exceeding the respective elimination thresholds in each EU.^10^

Tigray Regional Health Bureau (RHB) launched projects to eliminate trachoma as a public health problem in 2015. By 2018, 31 of the 34 trachoma-endemic woredas had completed the required number of rounds of antibiotic MDA and more than 38,000 TT surgeries had been performed. No trachoma-targeted facial cleanliness or environmental interventions were conducted during that period.

WHO recommends that trachoma impact surveys be undertaken 6−12 months after the last round of annual antibiotic MDA, to determine whether treatment should be continued or stopped.^18^ The primary aim of the surveys reported here was to re-estimate TF prevalence among children aged 1–9 years after interventions in the Tigray Region, Ethiopia, to guide future interventions. The prevalence of TT and water, sanitation, and hygiene (WaSH) infrastructure coverage was also assessed.

## Methods

### Survey overview

At the end of 2019, Tropical Data protocols were updated from version 2^19^ to version 3.^20^ Version 2 was used for the 2018–19 surveys, and version 3 for the 2020 surveys. Methodological differences are noted in the relevant sections below.

All 31 woredas were surveyed in the first series of surveys, and 11 woredas were resurveyed in the second. The first series of surveys in these woredas involved 22 surveys in 2018−2019 (using the Tropical Data version 2 methodology)^19^ and nine surveys in 2020 (using the Tropical Data version 3 methodology).^20^ Out of the 22 surveys conducted in 2018–2019, 21 were above the 5% WHO threshold. Out of these 21, 12 had a TF prevalence between 5.0% and 9.9% and therefore only required a single extra round of MDA, and were therefore available for a second series of impact surveys by 2020. The second series however consisted of surveys in only 11 of these woredas, which had completed planned MDA treatment as per WHO guidelines (all using the Tropical Data version 3 methodology),^20^ as repeat impact surveys in one woreda, Tsegede, were put on hold due to insecurity in the area. In total, 20 woredas were surveyed once and 11 woredas were surveyed twice.

### Survey design

All surveys followed contemporary WHO recommendations for community-based cross-sectional survey design.^6,21^ Each woreda was surveyed as an independent EU, in-line with WHO definitions of an EU for trachoma elimination purposes.^16^ Within each woreda, two-stage cluster sampling was employed. The primary sampling unit was the kushat, selected using random sampling with probability proportional to size. The secondary sampling unit was the household, selected using compact segment sampling. All residents aged ≥1 year in selected households were eligible to take part.

To estimate a prevalence of TF in 1–9-year-olds of 4% with a precision of ± 2% (at the 95% confidence level, assuming a design effect of 2.63^22^ and assuming a 20% non-response rate), 1,164 children aged 1–9 years were needed.^21^ Based on recent trachoma surveys in Ethiopia, an average of 1.5 children aged 1–9 years was expected per household, so 776 households would be needed to meet the sample size target. Based on sampling 30 households (the number which could comfortably be visited by one field team in a single day) per kushat, 26 kushats would need to be included. The secondary objectives of the survey (estimation of TT prevalence and WaSH coverage) would be assessed in the households selected to recruit children for TF assessment.

### Training and team composition

All graders and recorders were trained in a standardised manner using the Tropical Data training system.^19,20^ All had to pass a standard qualifying examination before they could join a survey team. Graders were required to pass both slide and field intergrader agreement tests by scoring kappas of ⩾.7 compared to a certified grader trainer, and recorders were required to score ≥90% in a standard recorder test.^19,20^

Thirteen teams, each consisting of a trachoma grader, a data recorder, a village guide, and a driver, were assembled to conduct the fieldwork. A supervisor oversaw the work of graders and recorders and assisted them technically, as well as coordinating and leading the overall survey.

### Clinical data collection

Field teams moved from house-to-house to collect the data. All residents aged ≥1 year living in selected households were invited to be examined. Children aged <1 year and individuals not resident in selected households (including institutionalised and homeless) were excluded.

Each consenting individual was examined by a certified grader for the signs TT, TF, and trachomatous inflammation—intense (TI),^23^ using 2.5× magnifying binocular loupes, and sunlight or a torch for illumination. In 2020, follicle size guides were used to support TF diagnosis.^24^ When trichiasis was recorded as being present in an eye, the presence or absence of trachomatous scarring (TS)^23^ was also checked, and the examinee was asked if they had ever been offered management of the trichiasis in that eye by a health-care worker at a primary, secondary, or tertiary level. All data were captured electronically using a purpose-built Open Data Kit-based Android smartphone application (www.tropicaldata.org).

TT was graded differently in the 2018–19 and 2020 surveys, due to an amendment to the simplified grading system.^25,26^ In 2018–19, TT was graded according to the 1987 definition: at least one eyelash touching the eyeball or evidence of recent removal of in-turned eyelashes arising from either eyelid.^23^ In 2020, the eyelid (upper or lower) that a deviated eyelash originated from was recorded and TT was only assigned if the deviated eyelash originated from the upper eyelid, in-line with contemporary recommendations.^25,26^

### Household data collection

The GPS coordinates of participating households were recorded and the household head or their nominee was asked questions about household WaSH access. Questions were adapted for trachoma surveys from the United Nations Children’s Fund (UNICEF)/WHO Joint Monitoring Programme (JMP) household questionnaire.^6,27,28^ Latrines and handwashing facilities were directly observed.

Handwash station assessment differed between 2018–19 and 2020. In 2018–19, only handwash stations within 15 m of the household latrine were recorded.^6,19,27^ Where there was no latrine, no handwash station data were recorded. In 2020, householders were asked whether there was a handwash station on the household premises, regardless of the presence or absence of a latrine.^20,28^

In households in which one or more resident 1–9-year-olds were missing at the time of the survey team’s first visit, a second visit was made, where possible, before the end of the day.

### Data analysis

Woreda-level prevalence estimates and confidence intervals were calculated using the methods used by the Global Trachoma Mapping Project.^6,29^ TF prevalence in children aged 1–9 years was adjusted for age in 1-year age-bands. TT (according to the definition at the time of the survey) unknown to the health system prevalence in those aged ≥15 years was adjusted for age and gender in 5-year age-bands.

### Survey ethics

Approval for the survey was obtained from the Tigray Regional Health Research Institution Ethics Committee. Tropical Data support for trachoma surveys was approved by the London School of Hygiene & Tropical Medicine Observational Ethics Committee (16105). Informed verbal consent was obtained from all individuals (or their parent/guardian for children aged <18 years) who participated in the survey and recorded in the data collection app. All participants found to have active trachoma were provided with 1% tetracycline eye ointment for 6 weeks free-of-charge and those with trichiasis were referred for appropriate management.

## Results

### Population surveyed

A total of 1,099 kushats, 32,989 households, and 135,758 individuals were enumerated across the 42 surveys (Supplementary Table 1). Of 135,758 people enumerated, 130,872 (96%) were examined by survey teams; 4,730 (3%) were absent and 154 (.1%) refused. Ninety-nine percent of enumerated 1–9-year-olds were examined and 94% of enumerated adults aged ≥15 years were examined. Of those examined, 72,902 (56%) were female.

### Active trachoma

Of 54,796 children aged 1–9 years examined across all 42 surveys, 5,156 (10%) had TF in one or both eyes (Supplementary Table 2).

In the first wave of the 31 surveys, the prevalence of TF in 1−9-year-olds ranged from 3.1% (95% confidence interval [CI]: 1.7−5.0%) in Tahtay Adiabo to 24.5% (95% CI: 19.9−29.0%) in Ofla. There were 1, 13, and 17 woredas with a TF prevalence of <5.0%, 5.0−9.9%, and 10.0−29.9%, respectively.

**Figure 1:**
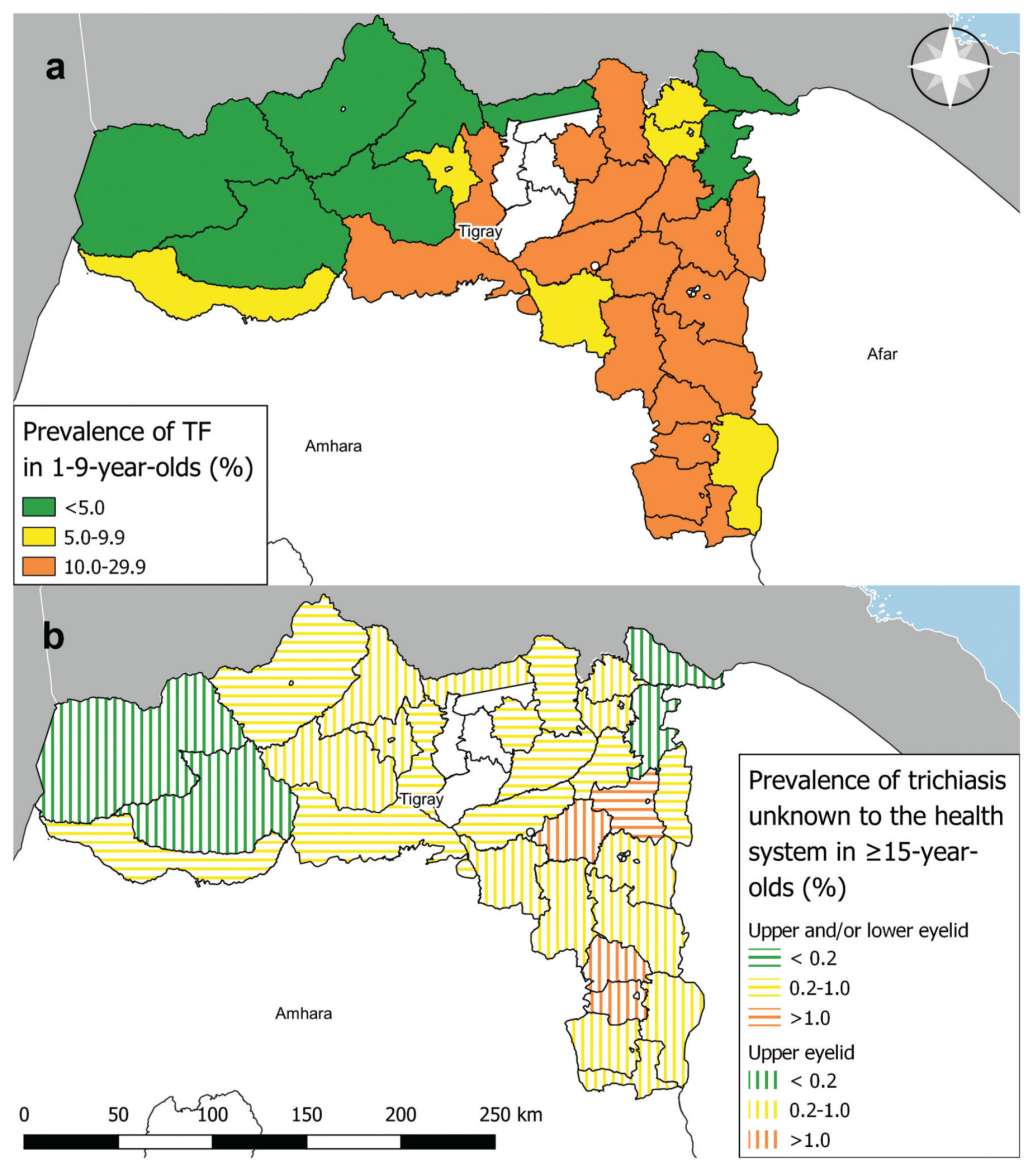
Trachoma impact survey results from most recent survey in 31 woredas of Tigray region, Ethiopia, 2018–2020. (a) age-adjusted prevalence of trachomatous inflammation—follicular (TF) in 1–9-year-olds. (b) age- and gender-adjusted prevalence of trachomatous trichiasis (TT) unknown to the health system in ≥15-year-olds, according to contemporary definitions at the time of the survey. The boundaries and names shown and the designations used on this map do not imply the expression of any opinion whatsoever on the part of the authors, or the institutions with which they are affiliated, concerning the legal status of any country, territory, city or area or of its authorities, or concerning the delimitation of its frontiers or boundaries.

**Figure 2:**
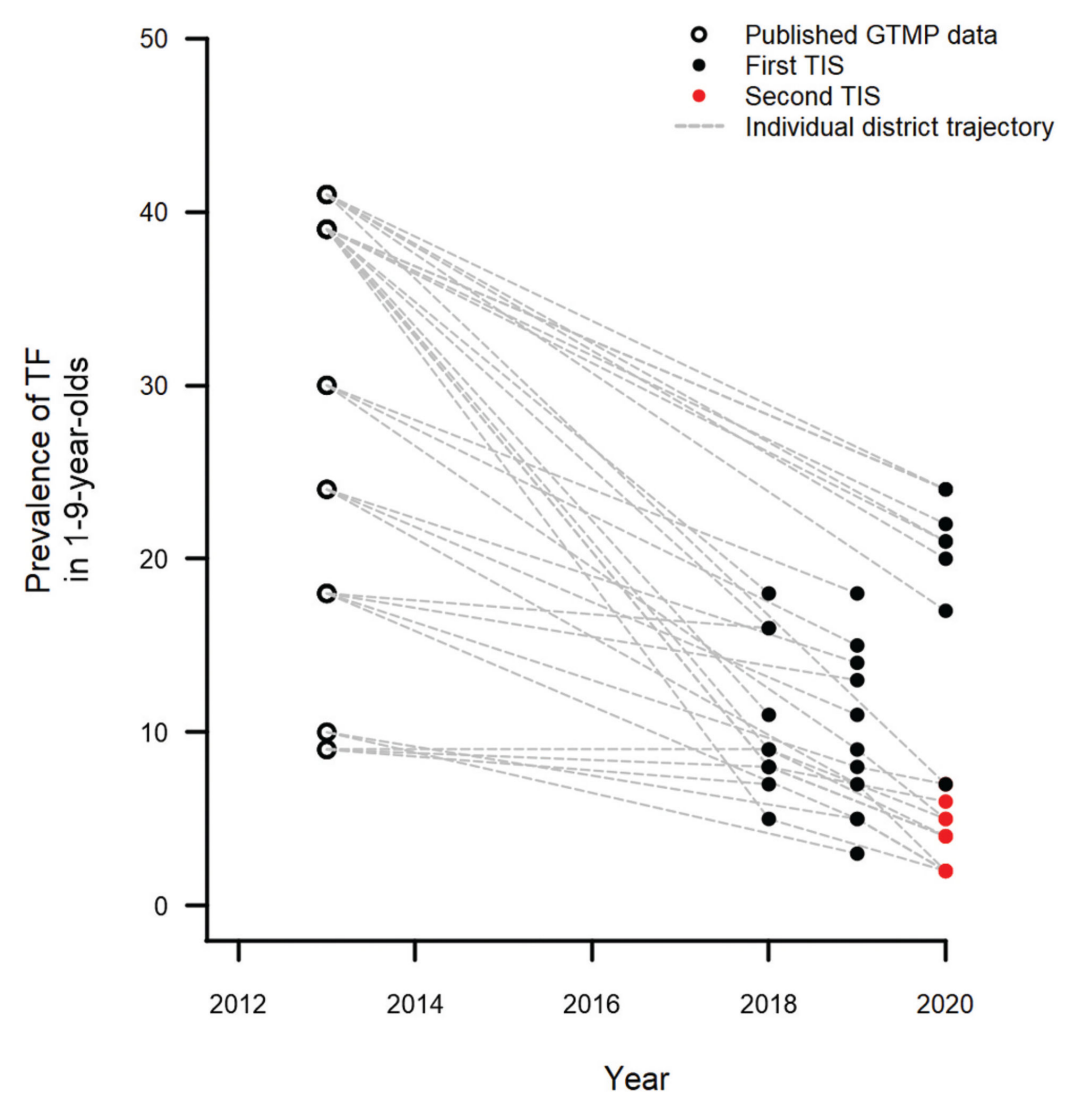
Prevalence of trachomatous inflammation—follicular (TF) before and after mass drug administration (MDA) in 31 woredas of Tigray region, Ethiopia. Pre-MDA prevalence estimates have been published previously.^10^ Confidence intervals are not presented to avoid overcomplicating the figure. This figure is for descriptive purposes only: because the pre- and post- MDA woreda boundaries are different, formal pre- and post-MDA estimate comparisons are not appropriate. GTMP: Global Trachoma Mapping Project; TIS: Trachoma Impact Survey.

Eleven of the 13 woredas with a TF prevalence 5.0−9.9% in the first series of surveys received one round of MDA and were re-surveyed in a second series of surveys. The prevalence of TF in 1–9-year-olds in this series ranged from 1.5% (95% CI: .7−2.4%) in Asgede Tsimbla to 7.2% (95% CI: 4.4−10.2%) in Tahtay Koraro. There were 7 and 4 woredas with a TF prevalence of <5.0% and 5.0−9.9%, respectively. Figure 1a shows the most recent estimates of TF prevalence from each woreda. Indicative changes in TF prevalence since baseline surveys are shown in Figure 2; it should be noted that, because the EU boundary has changed between pre-MDA and post-MDA surveys, these are for illustrative purposes only and not for formal comparisons.

### Trachomatous trichiasis

A total of 63,648 people aged ≥15 years were examined during all 42 surveys (Supplementary Table 3). During the first wave of surveys (22 surveys using the 2018−9 methodology surveys and 9 surveys using the 2020 methodology), 48228 ≥ 15-year-olds were examined. Eight hundred and ninety-one cases (621 [70%] unknown to the health system) of trichiasis (upper and/or lower eyelid) and 380 cases (261 [69%] unknown to the health system) of trichiasis (upper eyelid only) were identified. The prevalence of TT (upper and/or lower eyelid) unknown to the health system in ≥15-year-olds in the 2018−9 surveys in the first series ranged from .25% (95% CI: .04–.54%) in Tsegede to 1.15% (95% CI: .5−2.02%) in Kilte Awlaelo. The prevalence of TT (upper eyelid) unknown to the health system in ≥15-year-olds in the 2020 surveys in the first series ranged from .50% (95% CI: .26–.71%) in Enderta to 1.18% (95% CI: .78−1.61%) in Degua Temben. The prevalence of TT unknown to the health system was ≥ .2% in all 31 surveys. During the second series of surveys (11 surveys), 222 cases of TT (upper eyelid only) were identified, of which 127 [57%] were unknown to the health system. The prevalence of TT (upper eyelid only) unknown to the health system in ≥15-year-olds ranged from .12% (95% CI: .03 − .26%) in Erob to .55% (95% CI: .21 −1.00%) in Tahtay Koraro. The prevalence was ≥ .2% in 7/11 surveys. Figure 1b shows the most recent prevalence estimates of TT unknown to the health system, according to contemporary definitions at the time of the survey, in each woreda.

### Water, sanitation, and hygiene access

Overall, WaSH access was low (Supplementary Table 4). Of the 32,989 households surveyed in 1,099 kushats in 42 surveys, 9,949 (30%) had access to an improved drinking water source within a 30-minute return journey of the house and 3,632 (11%) had an improved latrine. There were 588/32,989 (2%) households with a handwashing facility across all 42 surveys. For the 22 surveys that used version 2 methodology, 300/17,431 households had a latrine with a handwashing station within 15 m of the latrine. For the 20 surveys that used the version 3 methodology, 288/15,558 had a handwashing station in the yard/plot/premises.

## Discussion

Following several years of MDA, a general decrease in TF prevalence has been observed as compared to pre-intervention estimates in Tigray, Ethiopia. However, WHO targets for elimination of trachoma as a public health problem were not met in 23 surveyed woredas, and ongoing interventions are required. Of these, 23 woredas that require further rounds of antibiotic MDA, six will need one round and 17 will need three rounds before resurvey. In eight woredas, the target TF prevalence has been reached. No further rounds of MDA should be delivered for 24 months before a trachoma surveillance survey is conducted. Four woredas, namely Erob, Saesi-Tsaedamba, Kafta-humera and Welkayit, met both TF and TT elimination criteria.

The decline in TF prevalence over the course of the programme has been slower than expected. While this is perhaps unsurprising given similar findings from neighbouring regions of Ethiopia,^5,30^ operational research to explore the fidelity of program implementation could help identify areas that could be improved to make MDA more locally effective. Additionally, antibiotic MDA alone will not break the cycle of *C. trachomatis* transmission: adequate WaSH infrastructure and behaviour change interventions are likely to be essential, alongside MDA, to achieving sustained elimination of trachoma.^31–33^ Sustained and extensive health education is required to promote and maintain behavioural changes in personal and environmental hygiene.^32,34^ Potential routes for intervention could be through integration with school health curricula and through health worker training packages. To facilitate behaviour change, WaSH infrastructure must be improved. There is a need for increased intersectoral collaboration between the neglected tropical disease and WaSH sectors, as well as increased financial resources for WaSH interventions in the context of trachoma elimination.^33,35^ Collection of WaSH access data within trachoma surveys enables planning of district-specific environmental improvement activities and advocacy for support from partner organisations. Furthermore, improvements in WaSH infrastructure will contribute to the achievement of wider national and international targets to reduce inequality.^36^

The prevalence of TT remains high in Tigray and above the elimination target in 27 woredas. The majority of TT cases identified in these surveys were previously unknown to the health system, indicating that significant improvements in case-finding and surgical service accessibility are needed. The link between TT and female gender is well established^37^; it is important to design surgical programmes to ensure women have equitable access to services. Improvements to surgical services should not just aim to increase coverage but also maintain and enhance surgical quality, and sustainably identify incident cases, to meet the WHO elimination criteria.^4^ Mechanisms to detect postoperative TT should be developed, and for patients who are not operated on at first diagnosis of TT^38,39^ but subsequently wish to have surgery,^40^ it should be readily available. The number, geographical distribution and motivation of surgeons, and their access to appropriate equipment, should be reviewed to identify areas for service improvement. Programmes should also work to increase awareness and uptake of TT surgery in affected communities.

We found that one-third of households had access to an improved drinking water source within a 30-minute return journey of the house. This is consistent with previous findings from Ethiopia. The UNICEF/WHO JMP, for example, estimated that in 2015, 30% of rural Ethiopian households had access to an improved drinking water source within a 30-minute return journey.^41^ However, our findings suggest that Tigray has lower WaSH access than other regions of Ethiopia due to failure to mobilise resources for construction of WaSH facilities. The 2016 Ethiopian Demographic and Health Survey estimated that 57% of households in rural areas had access to basic drinking water.^42^ Despite regional and national recommendations for householders to invest in solid covers for their own sanitation facilities, the majority of households that we visited (89%) did not have access to improved latrines. Of the eight woredas with a TF prevalence below the 5% threshold, the WaSH ratios were not observed to be higher than those that had not reached elimination.

These surveys were technically strong. The adherence to internationally standardised survey protocols and the apparently high examination coverage of residents in selected households suggest that the data are robust and comparable to trachoma data from elsewhere. There were also some weaknesses in our approach. First, the surveys were designed to estimate TF prevalence and may be under-powered to precisely estimate TT prevalence: the number of adults examined was below the 2,818 sample size recommended by WHO^43^ in all 42 surveys. We have ensured confidence intervals around TT prevalence estimates are presented clearly alongside point estimates. Second, the EU boundaries of the baseline surveys do not match those used here. It is therefore not possible to make formal comparisons between pre- and post-MDA data and any comparisons, such as those we have drawn in Figure 2, are for indicative purposes only. The primary application of these data is for planning future interventions, for which they are critical;^44,45^ this does not rely on comparisons to previous prevalence estimates.

There is still an appreciable programme requirement in Tigray with 23 woredas requiring further MDA and further impact surveys and eight woredas requiring surveillance surveys in 2 years’s time. Since these surveys took place, the trachoma programme has been impacted initially by the global coronavirus pandemic and then by conflict in the region. For these reasons, planned antibiotic MDA and survey activities had been on hold for 2 years at the time of publication. The impact of this interruption on disease prevalence will not be clear until activities resume. The conflict has impacted other parts of the trachoma programme. TT surgical services have continued during this period, but their capacity has been restricted by the local instability. Medical supplies have been very limited, necessitating a shift from outreach to static clinics. A recent eye health service readiness assessment in Tigray demonstrated physical damage to health posts and lack of basic (such as dosing pole, registration books, and drugs) or surgical equipment. More general resources such as fuel, communications, and banking services are also unavailable as a result of the conflict. Staff salaries have been paid despite absence of materials to conduct programmatic activities, leading to ongoing costs despite interruption of activities.

## Conclusions

The prevalence of trachoma in the Tigray region is still high, with the TF elimination threshold having been reached in only eight woredas. To eliminate trachoma from Tigray, adequate human, financial, and material resources should be mobilised to strengthen ongoing SAFE implementation and periodic monitoring, evaluation, and reporting of progress as per WHO recommendations.

## Supplementary Material

Supplemental data for this article can be accessed online at https://doi.org/10.1080/09286586.2024.2317823.

Supplemental material
