# Supplementary material for: Trachoma Impact Survey Results from 31 Woredas in Tigray Region, Ethiopia
Source: Ophthalmic Epidemiol. Author manuscript; Available in PMC 2026 Feb 26. (PMC7618801; doi:10.1080/09286586.2024.2317823)
Supplement: Supplemental material [file EMS212682-supplement-Supplemental_material.docx]

**Supplementary material for:**

**Trachoma impact survey results from 31 woredas in Tigray Region, Ethiopia.**

Gemechis Teferi^1^*, Harnet Adane^2^, Evini Cyrille^1^, Aynalem Tefera^1^, Solomon Gadisa^1^, Adugna Amin^1^, Mebratu Tsehaye^1^, Yonas Mitku^3^, Haftamu Assefa^3^, Sharone Backers^4^, Addisu Alemayehu^4^, Belete Mengistu^4^, Fikreab Kebede^5^, Fentahun Tadesse^5,6^, Nebiyu Nigusse^5^, Robert Butcher^7^, Ana Bakhtiari^8^, Rebecca Willis^8^, Sarah Boyd^8^, Cristina Jimenez^9^, Michael Dejene^10^, Anthony W. Solomon^11^, Meheret Deyassa^12^, Mohammed Shafi^13^, Tezera Kifle^14^, Asfaw Tegen^15^, Berihu Mesfin^2^, Tsegay Berihu^2^, Teklay Mariam^2^, Hagos Godefay^2^, Emma M. Harding-Esch^7^, Amanuel Kidane^1,^ Ephrem Fisseha^1^.

Affiliations

1. Light for the World, Ethiopia
2. Tigray Regional Health Bureau, Ethiopia
3. Quha Hospital, Tigray, Ethiopia
4. Act to End NTDs East, RTI International, Addis Ababa, Ethiopia
5. Ministry of Health, Ethiopia
6. Crown Agents
7. Clinical Research Department, London School of Hygiene & Tropical Medicine, UK
8. Task Force for Global Health, Decatur, GA, USA
9. Sightsavers, Haywards Heath, UK
10. Sightsavers, Addis Ababa, Ethiopia
11. Department of Control of Neglected Tropical Diseases, World Health Organization, Geneva, Switzerland
12. Ambo Hospital, Ethiopia
13. Adama Hospital Medical College, Ethiopia
14. Orbis International, Ethiopia
15. Eyen Consulting PLC, Ethiopia

# **Table 1.** Summary of the population enumerated and examined in trachoma impact surveys in 31 woredas of Tigray region, Ethiopia, 2018–2020.

| **Woreda**  **(Evaluation Unit ID)** | **Date survey completed** | **Population ≥1 year enumerated** | **Population ≥1 year absent** | **Population ≥1 year refused** | **Population ≥1 year examined (%)** | **Population ≥1 year examined who were female (%)** | **Population aged ≥15 years enumerated** | **Children aged 1-9 years enumerated** | **Proportion of ≥15 year-olds examined (%)** | **Proportion of 1-9 year-olds examined (%)** |
| --- | --- | --- | --- | --- | --- | --- | --- | --- | --- | --- |
| Mereb Leke (80910) | Jan-2019 | 3,351 | 78 | 0 | 3,273 (98) | 1,707 (52) | 1,749 | 1,280 | 1,686 (96) | 1,279 (>99) |
| Mereb Leke (81258) | Mar-2020 | 3,038 | 34 | 2 | 3,002 (99) | 1,739 (58) | 1,426 | 1,320 | 1,395 (98) | 1,317 (>99) |
| Tanqua Abergele (80911) | Jan-2019 | 3,455 | 38 | 0 | 3,417 (99) | 1,729 (51) | 1,783 | 1,359 | 1,755 (98) | 1,355 (>99) |
| Tanqua Abergele (81259) | Mar-2020 | 3,147 | 58 | 2 | 3,087 (98) | 1,772 (57) | 1,421 | 1,423 | 1,374 (97) | 1,420 (>99) |
| Erob (80914) | Dec-2018 | 3,254 | 213 | 0 | 3,041 (93) | 1,667 (55) | 1,693 | 1,244 | 1,516 (90) | 1,237 (99) |
| Erob (81260) | Apr-2020 | 2,865 | 29 | 2 | 2,834 (99) | 1,563 (55) | 1,461 | 1,167 | 1,434 (98) | 1,165 (>99) |
| Ganta Afeshum (80915) | Dec-2018 | 3,261 | 163 | 0 | 3,098 (95) | 1,713 (55) | 1,757 | 1,225 | 1,628 (93) | 1,218 (99) |
| Ganta Afeshum (81261) | Mar-2020 | 3,006 | 71 | 0 | 2,935 (98) | 1,659 (57) | 1,403 | 1,365 | 1,364 (97) | 1,346 (99) |
| Glomekeda (80916) | Dec-2018 | 3,375 | 187 | 0 | 3,188 (94) | 1,701 (53) | 1,760 | 1,293 | 1,620 (92) | 1,286 (99) |
| Glomekeda (81262) | Mar-2020 | 3,047 | 51 | 1 | 2,995 (98) | 1,665 (56) | 1,486 | 1,318 | 1,447 (97) | 1,312 (>99) |
| Saesi Tsaeda Amba (80919) | Dec-2018 | 3,423 | 315 | 6 | 3,102 (91) | 1,730 (56) | 1,770 | 1,205 | 1,486 (84) | 1,203 (>99) |
| Saesi Tsaeda Amba (81263) | Mar-2020 | 3,053 | 79 | 3 | 2,971 (97) | 1,698 (57) | 1,450 | 1,359 | 1,403 (97) | 1,345 (99) |
| Asgede Tsimbla (80920) | Jan-2019 | 3,594 | 133 | 0 | 3,461 (96) | 1,867 (54) | 1,813 | 1,361 | 1,717 (95) | 1,347 (99) |
| Asgede Tsimbla (81264) | Mar-2020 | 3,097 | 47 | 10 | 3,040 (98) | 1,775 (58) | 1,449 | 1,339 | 1,395 (96) | 1,339 (100) |
| Laelay Adyabo (80921) | Jan-2019 | 3,460 | 98 | 0 | 3,362 (97) | 1,831 (54) | 1,779 | 1,308 | 1,702 (96) | 1,306 (>99) |
| Laelay Adyabo (81265) | Mar-2020 | 3,011 | 66 | 10 | 2,934 (97) | 1,759 (60) | 1,422 | 1,339 | 1,350 (95) | 1,337 (>99) |
| Tahtay Koraro (80924) | Jan-2019 | 3,370 | 53 | 1 | 3,316 (98) | 1,792 (54) | 1,742 | 1,307 | 1,701 (98) | 1,302 (>99) |
| Tahtay Koraro (81266) | Mar-2020 | 3,161 | 58 | 5 | 3,098 (98) | 1,763 (57) | 1,490 | 1,345 | 1,433 (96) | 1,343 (>99) |
| Degua Temben (81267) | Feb-2020 | 3,164 | 86 | 11 | 3,067 (97) | 1,754 (57) | 1,432 | 1,378 | 1,366 (95) | 1,365 (99) |
| Enderta (81268) | Feb-2020 | 3,182 | 174 | 13 | 2,995 (94) | 1,728 (58) | 1,513 | 1,267 | 1,375 (91) | 1,244 (98) |
| Hintalo-Wojerat (81269) | Feb-2020 | 3,147 | 84 | 7 | 3,056 (97) | 1,784 (58) | 1,477 | 1,357 | 1,411 (96) | 1,346 (99) |
| Sahrti-Samre (81270) | Mar-2020 | 3,220 | 56 | 5 | 3,159 (98) | 1,792 (57) | 1,477 | 1,444 | 1,436 (97) | 1,434 (99) |
| Alaje (81271) | Feb-2020 | 2,937 | 62 | 1 | 2,874 (98) | 1,677 (58) | 1,366 | 1,361 | 1,318 (96) | 1,355 (>99) |
| Alamata (81272) | Feb-2020 | 3,024 | 45 | 11 | 2,968 (98) | 1,715 (58) | 1,409 | 1,328 | 1,370 (97) | 1,319 (99) |
| Enda mohoni (81273) | Feb-2020 | 2,999 | 48 | 6 | 2,945 (98) | 1,668 (57) | 1,420 | 1,373 | 1,379 (97) | 1,365 (99) |
| Ofla (81274) | Feb-2020 | 2,892 | 51 | 6 | 2,834 (98) | 1,654 (58) | 1,366 | 1,304 | 1,320 (97) | 1,297 (99) |
| Raya Azebo (81275) | Feb-2020 | 3,041 | 55 | 5 | 2,981 (98) | 1,759 (59) | 1,401 | 1,388 | 1,361 (97) | 1,380 (99) |
| Kafta Humera (80926) | Dec-2018 | 3,500 | 145 | 1 | 3,354 (96) | 1,824 (54) | 1,708 | 1,404 | 1,595 (93) | 1,398 (>99) |
| Kafta Humera (81276) | Mar-2020 | 3,196 | 44 | 7 | 3,145 (98) | 1,777 (57) | 1,491 | 1,348 | 1,443 (97) | 1,348 (100) |
| Welkayit (80928) | Dec-2018 | 3,326 | 72 | 2 | 3,252 (98) | 1,796 (55) | 1,643 | 1,337 | 1,576 (96) | 1,336 (>99) |
| Welkayit (81278) | Apr-2020 | 3,023 | 34 | 5 | 2,984 (99) | 1,737 (58) | 1,419 | 1,346 | 1,382 (97) | 1,345 (>99) |
| Ahferom (80908) | Jan-2019 | 3,386 | 85 | 1 | 3,300 (97) | 1,762 (53) | 1,769 | 1,290 | 1,710 (97) | 1,284 (>99) |
| Kolla Temben (80909) | Jan-2019 | 3,260 | 51 | 0 | 3,209 (98) | 1,718 (54) | 1,686 | 1,295 | 1,647 (98) | 1,295 (100) |
| Werehilehi (80912) | Jan-2019 | 3,244 | 70 | 1 | 3,173 (98) | 1,648 (52) | 1,707 | 1,272 | 1,650 (97) | 1,269 (>99) |
| Atsibi Wonberta (80913) | Dec-2018 | 3,368 | 422 | 11 | 2,935 (87) | 1,761 (60) | 1,964 | 950 | 1,625 (83) | 923 (97) |
| Hawzien (80917) | Dec-2018 | 3,466 | 296 | 4 | 3,166 (91) | 1,769 (56) | 1,797 | 1,333 | 1,553 (86) | 1,320 (99) |
| Kilte Awlaelo (80918) | Nov-2018 | 3,299 | 611 | 14 | 2,674 (81) | 1,645 (62) | 1,888 | 1,001 | 1,407 (75) | 955 (95) |
| Medebay Zana (80922) | Jan-2019 | 3,353 | 74 | 0 | 3,279 (98) | 1,745 (53) | 1,745 | 1,294 | 1,684 (97) | 1,294 (100) |
| Tahtay adiabo (80923) | Jan-2019 | 3,537 | 90 | 0 | 3,447 (97) | 1,819 (53) | 1,755 | 1,421 | 1,688 (96) | 1,415 (>99) |
| Tselemti (80925) | Dec-2018 | 3,499 | 94 | 0 | 3,405 (97) | 1,800 (53) | 1,765 | 1,370 | 1,693 (96) | 1,360 (99) |
| Tsegede (80927) | Dec-2018 | 3,330 | 132 | 0 | 3,198 (96) | 1,744 (55) | 1,605 | 1,433 | 1,519 (95) | 1,426 (>99) |
| Adwa (80907) | Jan-2019 | 3,397 | 78 | 1 | 3,318 (98) | 1,696 (51) | 1797 | 1,269 | 1,734 (96) | 1,266 (>99) |
|  |  |  |  |  |  |  |  |  |  |  |

# **Table 2.** Post-antibiotic mass drug administration prevalence of trachomatous inflammation—follicular (TF) in 31 woredas of Tigray region, Ethiopia, trachoma impact surveys, 2018–2020.

| **Woreda** | **Year of survey** | **Number of 1–9-year-olds examined** | **Number of children aged 1–9 years with TF** | **Number of children aged 1–9 years with TI** | **Age-adjusted prevalence of TF in 1–9-year-olds** (95% CI) |
| --- | --- | --- | --- | --- | --- |
| Mereb Leke | 2019 | 1,279 | 83 | 9 | 7.3 (4.5-10.9) |
|  | 2020 | 1,317 | 24 | 7 | 2.4 (1.2-3.8) |
| Tanqua Abergele | 2019 | 1,355 | 113 | 9 | 9.4 (6.2-12.5) |
|  | 2020 | 1,420 | 67 | 8 | 5.1 (2.7-8.0) |
| Erob | 2018 | 1,237 | 58 | 6 | 5.1 (2.8-7.3) |
|  | 2020 | 1,165 | 19 | 7 | 1.8 (0.7-2.8) |
| Ganta Afeshum | 2018 | 1,218 | 91 | 10 | 7.8 (4.1-12.2) |
|  | 2020 | 1,346 | 78 | 12 | 6.0 (3.0-9.9) |
| Glomekeda | 2018 | 1,286 | 115 | 9 | 9.1 (5.9-12.4) |
|  | 2020 | 1,312 | 60 | 8 | 5.2 (3.2-7.9) |
| Saesi Tsaeda Amba | 2018 | 1,203 | 102 | 7 | 8.3 (5.1-11.0) |
|  | 2020 | 1,345 | 59 | 8 | 4.2 (2.2-6.8) |
| Asgede Tsimbla | 2019 | 1,347 | 61 | 11 | 5.0 (2.6-7.8) |
|  | 2020 | 1,339 | 20 | 12 | 1.5 (0.7-2.4) |
| Laelay Adyabo | 2019 | 1,306 | 61 | 9 | 5.1 (2.8-8.0) |
|  | 2020 | 1,337 | 24 | 3 | 2.0 (1.0-3.1) |
| Tahtay Koraro | 2019 | 1,302 | 85 | 14 | 7.6 (5.0-10.6) |
|  | 2020 | 1,343 | 83 | 33 | 7.2 (4.4-10.2) |
| DeguaTemben | 2020 | 1,365 | 288 | 24 | 23.6 (18.6-29.3) |
| Enderta | 2020 | 1,244 | 256 | 12 | 23.7 (18.9-28.4) |
| Hintalo-Wojerat | 2020 | 1,346 | 270 | 17 | 21.8 (17.9-26.9) |
| Sahrti-Samre | 2020 | 1,434 | 278 | 31 | 20.5 (16.4-25.8) |
| Alaje | 2020 | 1,355 | 271 | 28 | 20.8 (16.2-26.0) |
| Alamata | 2020 | 1,319 | 197 | 22 | 16.7 (11.6-23.0) |
| Enda mohoni | 2020 | 1,365 | 243 | 28 | 19.7 (14.1-25.9) |
| Ofla | 2020 | 1,297 | 297 | 35 | 24.5 (19.9-29.0) |
| Raya Azebo | 2020 | 1,380 | 95 | 10 | 7.0 (5.4-9.2) |
| Kafta Humera | 2018 | 1,398 | 112 | 9 | 9.1 (6.0-13.0) |
|  | 2020 | 1,348 | 50 | 7 | 3.9 (1.7-6.1) |
| Welkayit | 2018 | 1,336 | 96 | 8 | 8.1 (4.7-11.5) |
|  | 2020 | 1,345 | 51 | 9 | 3.6 (1.9-5.0) |
| Ahferom | 2019 | 1,284 | 155 | 15 | 13.9 (8.7-20.0) |
| KollaTemben | 2019 | 1,295 | 205 | 18 | 18.2 (12.9-24.2) |
| Werehilehi | 2019 | 1,269 | 147 | 21 | 15.2 (11.0-20.0) |
| Atsibi Wonberta | 2018 | 923 | 141 | 16 | 16.0 (11.1-20.2) |
| Hawzien | 2018 | 1,320 | 143 | 19 | 11.4 (7.3-15.8) |
| Kilte Awlaelo | 2018 | 955 | 205 | 14 | 18.4 (13.7-22.7) |
| Medebay Zana | 2019 | 1,294 | 145 | 12 | 13.1 (9.3-17.2) |
| Tahtay Adiabo | 2019 | 1,415 | 38 | 2 | 3.1 (1.7-5.0) |
| Tselemti | 2018 | 1,360 | 182 | 18 | 15.7 (10.6-20.0) |
| Tsegede | 2018 | 1,426 | 88 | 5 | 7.2 (4.0-10.1) |
| Adwa | 2019 | 1,266 | 116 | 13 | 10.9 (6.9-16.1) |
| *Total* |  | *54,796* | *5,272* | *575* | *-* |
| *CI: confidence interval; TI: Trachomatous inflammation─intense.* | | | | | |

# **Table 3.** Age- and gender-adjusted prevalence of trachomatous trichiasis (TT) unknown to the health system in ≥15-year-olds in 31 woredas of Tigray region, Ethiopia, trachoma impact surveys, 2018–2020.

| **Woreda** | **Year of survey** | **Number of adults aged ≥15 years examined** | **Number of ≥15-year-olds with TT (upper and/or lower eyelid)** | **Number of ≥15-year-olds with TT (upper and/or lower eyelid) unknown to the health system** | **Adjusted* prevalence of TT (upper and/or lower eyelid) unknown to the health system in those aged≥15 years (95% CI)** | **Number of ≥15-year-olds with TT (upper eyelid only)** | **Number of ≥15-year-olds with TT (upper eyelid only) unknown to the health system** | **Adjusted* prevalence of TT (upper eyelid only) unknown to the health system in those aged≥15 years (95% CI)** |  |  |
| --- | --- | --- | --- | --- | --- | --- | --- | --- | --- | --- |
| Mereb Leke | 2019 | 1,686 | 30 | 22 | 0.55 (0.27-0.90) |  |  |  |  |  |
|  | 2020 | 1,395 |  |  |  | 18 | 10 | 0.25 (0.06-0.52) |  |  |
| Tanqua Abergele | 2019 | 1,755 | 32 | 27 | 0.64 (0.30-0.95) |  |  |  |  |  |
|  | 2020 | 1,374 |  |  |  | 21 | 17 | 0.45 (0.17-0.81) |  |  |
| Erob | 2018 | 1,516 | 20 | 16 | 0.55 (0.19-1.07) |  |  |  |  |  |
|  | 2020 | 1,434 |  |  |  | 7 | 4 | 0.12 (0.03-0.26) |  |  |
| Ganta Afeshum | 2018 | 1,628 | 51 | 33 | 0.79 (0.32-1.52) |  |  |  |  |  |
|  | 2020 | 1,364 |  |  |  | 29 | 15 | 0.32 (0.15-0.51) |  |  |
| Glomekeda | 2018 | 1,620 | 57 | 36 | 0.75 (0.42-1.19) |  |  |  |  |  |
|  | 2020 | 1,447 |  |  |  | 46 | 26 | 0.48 (0.27-0.74) |  |  |
| Saesi Tsaeda Amba | 2018 | 1,486 | 47 | 36 | 1.13 (0.43-2.03) |  |  |  |  |  |
|  | 2020 | 1,403 |  |  |  | 19 | 9 | 0.17 (0.07-0.29) |  |  |
| Asgede Tsimbla | 2019 | 1,717 | 42 | 35 | 0.98 (0.58-1.48) |  |  |  |  |  |
|  | 2020 | 1,395 |  |  |  | 11 | 8 | 0.24 (0.06-0.49) |  |  |
| LaelayAdyabo | 2019 | 1,702 | 42 | 30 | 0.88 (0.45-1.42) |  |  |  | |  |
|  | 2020 | 1,350 |  |  |  | 24 | 14 | 0.45 (0.18-0.86) |  |  |
| TahtayKoraro | 2019 | 1,701 | 52 | 36 | 1.13 (0.59-1.82) |  |  |  | |  |
|  | 2020 | 1,433 |  |  |  | 28 | 15 | 0.55 (0.21-1.00) |  |  |
| Degua Temben | 2020 | 1,366 |  |  |  | 55 | 42 | 1.18 (0.78-1.61) |  |  |
| Enderta | 2020 | 1,375 |  |  |  | 35 | 23 | 0.50 (0.26-0.71) |  |  |
| Hintalo-Wojerat | 2020 | 1,411 |  |  |  | 40 | 31 | 0.78 (0.49-1.15) |  |  |
| Sahrti-Samre | 2020 | 1,436 |  |  |  | 36 | 26 | 0.72 (0.36-1.20) |  |  |
| Alaje | 2020 | 1,318 |  |  |  | 54 | 29 | 1.17 (0.54-1.93) |  |  |
| Alamata | 2020 | 1,370 |  |  |  | 48 | 36 | 0.98 (0.59-1.55) |  |  |
| Enda mohoni | 2020 | 1,379 |  |  |  | 45 | 32 | 1.02 (0.56-1.64) |  |  |
| Ofla | 2020 | 1,320 |  |  |  | 40 | 28 | 0.96 (0.57-1.36) |  |  |
| Raya Azebo | 2020 | 1,361 |  |  |  | 27 | 14 | 0.53 (0.28-0.83) |  |  |
| KaftaHumera | 2018 | 1,595 | 19 | 16 | 0.67 (0.27-1.19) |  |  |  | |  |
|  | 2020 | 1,443 |  |  |  | 7 | 3 | 0.13 (0.00-0.32) |  |  |
| Welkayit | 2018 | 1,576 | 17 | 16 | 0.54 (0.25-0.88) |  |  |  | | |
|  | 2020 | 1,382 |  |  |  | 12 | 6 | 0.16 (0.03-0.37) |  |  |
| Ahferom | 2019 | 1,710 | 38 | 28 | 0.67 (0.35-1.04) |  |  |  |  |  |
| KollaTemben | 2019 | 1,647 | 51 | 28 | 0.94 (0.58-1.4) |  |  |  |  |  |
| Werehilehi | 2019 | 1,650 | 46 | 28 | 0.53 (0.33-0.79) |  |  |  |  |  |
| AtsibiWonberta | 2018 | 1,625 | 31 | 21 | 0.52 (0.24-0.89) |  |  |  |  |  |
| Hawzien | 2018 | 1,553 | 57 | 40 | 1.00 (0.67-1.4) |  |  |  |  |  |
| KilteAwlaelo | 2018 | 1,407 | 79 | 49 | 1.15 (0.5-2.02) |  |  |  |  |  |
| Medebay Zana | 2019 | 1,684 | 45 | 30 | 0.86 (0.48-1.34) |  |  |  |  |  |
| Tahtayadiabo | 2019 | 1,688 | 23 | 14 | 0.67 (0.31-1.15) |  |  |  |  |  |
| Tselemti | 2018 | 1,693 | 50 | 40 | 1.00 (0.62-1.45) |  |  |  |  |  |
| Tsegede | 2018 | 1,519 | 8 | 6 | 0.25 (0.04-0.54) |  |  |  |  |  |
| Adwa | 2019 | 1,734 | 54 | 34 | 0.86 (0.47-1.37) |  |  |  |  |  |
| *Total* |  | *63,648* | *891* | *621* |  | *602* | *388* | *-* |  |  |
| * For age and gender in five-year age bands, according to the most recent census.^45^  *CI: confidence interval* | | | | | | | | |  |  |

# **Table 4.** Proportion of households with adequate water, sanitation and hygiene access in 31 woredas of Tigray region, Ethiopia, trachoma impact surveys, 2018–2020.

| **Woreda** | **Date of surveyed year** | **Number of kushats visited** | **Number of households visited** | **Number of households with an improved drinking water source within 30 minutes of the house (%)** | **Number of households with an improved latrine (%)** | **Number of households with a latrine with a handwash station (%)** | **Number of households with a handwash station on the premises (%)** |
| --- | --- | --- | --- | --- | --- | --- | --- |
| Mereb Leke | 2019 | 26 | 779 | 312 (40) | 100 (13) | 1 (<1) |  |
|  | 2020 | 26 | 780 | 309 (40) | 130 (17) |  | 1 (<1) |
| Tanqua Abergele | 2019 | 26 | 778 | 96 (12) | 22 (3) | 0 (0) |  |
|  | 2020 | 26 | 781 | 111 (14) | 38 (5) |  | 11 (1) |
| Erob | 2018 | 26 | 786 | 196 (25) | 153 (19) | 41 (5) |  |
|  | 2020 | 25 | 750 | 186 (25) | 156 (21) |  | 63 (8) |
| Ganta Afeshum | 2018 | 26 | 781 | 217 (28) | 154 (20) | 20 (3) |  |
|  | 2020 | 26 | 781 | 209 (27) | 139 (18) |  | 1 (<1) |
| Glomekeda | 2018 | 26 | 778 | 257 (33) | 194 (25) | 51 (7) |  |
|  | 2020 | 26 | 781 | 219 (28) | 231 (30) |  | 3 (<1) |
| Saesi Tsaeda Amba | 2018 | 26 | 783 | 283 (36) | 35 (4) | 17 (2) |  |
|  | 2020 | 26 | 780 | 237 (30) | 98 (13) |  | 4 (1) |
| Asgede Tsimbla | 2019 | 26 | 786 | 174 (22) | 38 (5) | 0 (0) |  |
|  | 2020 | 26 | 781 | 145 (19) | 49 (6) |  | 2 (<1) |
| Laelay Adyabo | 2019 | 26 | 782 | 250 (32) | 66 (8) | 3 (<1) |  |
|  | 2020 | 26 | 777 | 184 (24) | 105 (14) |  | 2 (<1) |
| Tahtay Koraro | 2019 | 26 | 780 | 294 (38) | 73 (9) | 1 (<1) |  |
|  | 2020 | 26 | 778 | 217 (28) | 151 (19) |  | 2 (<1) |
| Degua Temben | 2020 | 26 | 779 | 160 (21) | 42 (5) |  | 1 (<1) |
| Enderta | 2020 | 26 | 778 | 134 (17) | 73 (9) |  | 8 (1) |
| Hintalo-Wojerat | 2020 | 26 | 778 | 257 (33) | 77 (10) |  | 9 (1) |
| Sahrti-Samre | 2020 | 26 | 779 | 193 (25) | 72 (9) |  | 6 (1) |
| Alaje | 2020 | 26 | 782 | 322 (41) | 65 (8) |  | 5 (1) |
| Alamata | 2020 | 26 | 777 | 364 (47) | 137 (18) |  | 4 (1) |
| Enda mohoni | 2020 | 26 | 780 | 180 (23) | 29 (4) |  | 6 (1) |
| Ofla | 2020 | 26 | 777 | 216 (28) | 48 (6) |  | 0 (0) |
| Raya Azebo | 2020 | 26 | 779 | 319 (41) | 130 (17) |  | 10 (1) |
| Kafta Humera | 2018 | 26 | 783 | 441 (56) | 66 (8) | 14 (2) |  |
|  | 2020 | 26 | 780 | 286 (37) | 121 (16) |  | 143 (18) |
| Welkayit | 2018 | 26 | 785 | 306 (39) | 50 (6) | 12 (2) |  |
|  | 2020 | 26 | 780 | 155 (20) | 44 (6) |  | 7 (1) |
| Ahferom | 2019 | 26 | 784 | 326 (42) | 110 (14) | 13 (2) |  |
| Kolla Temben | 2019 | 26 | 780 | 106 (14) | 23 (3) | 2 (<1) |  |
| Werehilehi | 2019 | 26 | 780 | 299 (38) | 98 (13) | 1 (<1) |  |
| Atsibi Wonberta | 2018 | 30 | 903 | 401 (44) | 63 (7) | 29 (3) |  |
| Hawzien | 2018 | 26 | 779 | 224 (29) | 77 (10) | 24 (3) |  |
| Kilte Awlaelo | 2018 | 30 | 901 | 366 (41) | 108 (12) | 44 (5) |  |
| Medebay Zana | 2019 | 26 | 779 | 172 (22) | 64 (8) | 9 (1) |  |
| Tahtayadiabo | 2019 | 26 | 780 | 157 (20) | 49 (6) | 1 (<1) |  |
| Tselemti | 2018 | 26 | 782 | 112 (14) | 42 (5) | 9 (1) |  |
| Tsegede | 2018 | 26 | 781 | 335 (43) | 80 (10) | 8 (1) |  |
| Adwa | 2019 | 26 | 781 | 222 | 32 (4) | 0 |  |
| *Total* |  | *1,099* | *32,989* | *9,949* | *3,632* | *300* | *288* |
